# Supplementary material for: Serum NMR-Based Metabolomics Profiling Identifies Lipoprotein Subfraction Variables and Amino Acid Reshuffling in Myeloma Development and Progression
Source: Int J Mol Sci. 2023 Jul 31;24(15):12275. doi: 10.3390/ijms241512275 (PMC10419104; doi:10.3390/ijms241512275)

Figure S1. Mean NMR NOESY spectra from control, MM and MGUS groups.

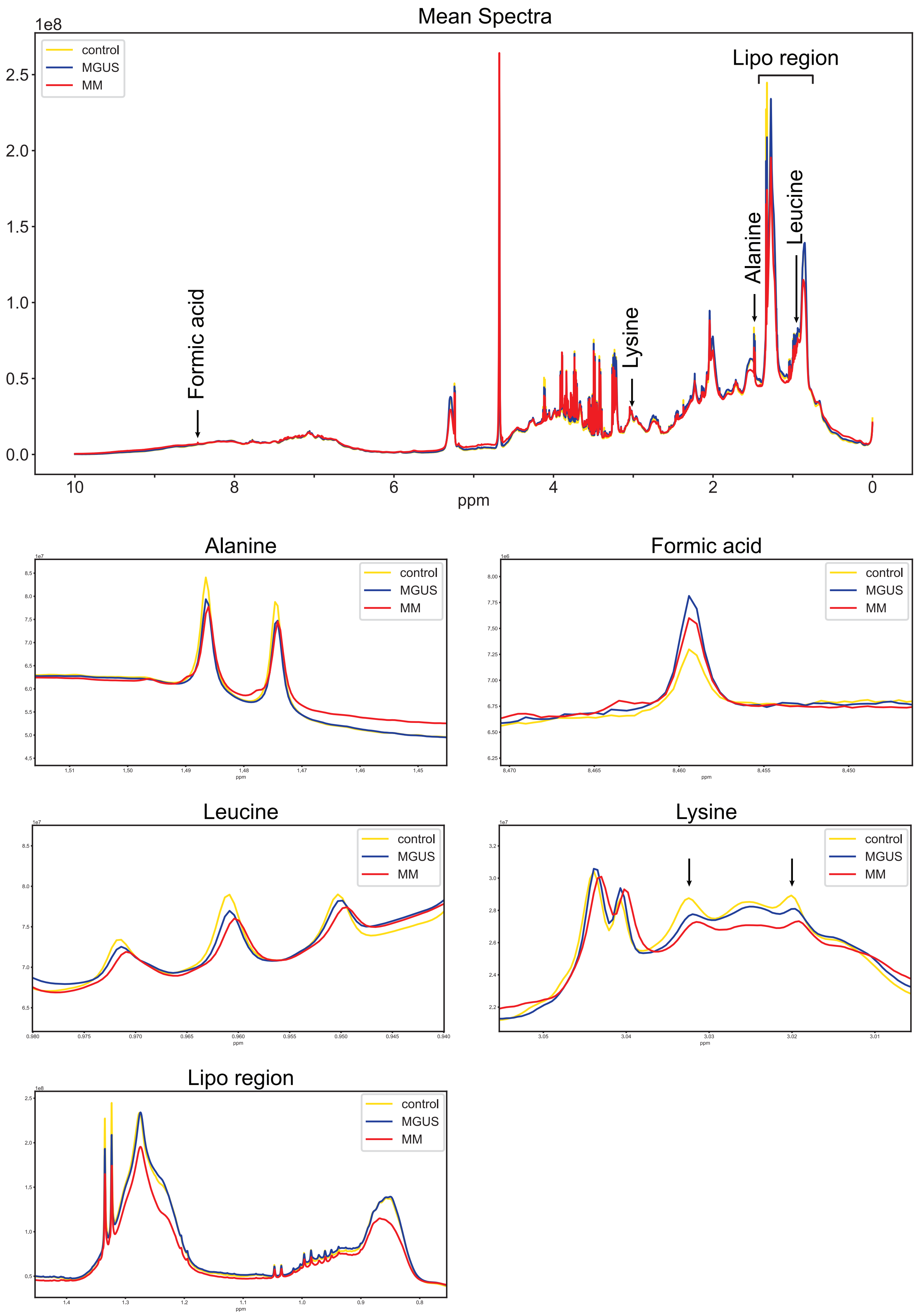

Figure S1. Mean NMR NOESY spectra from control, MM and MGUS groups. For easier visual comparison of alanine, formic acid, leucine and lysine levels, the baselines have been adjusted. The lipo region contains lipid signals used for lipoprotein subclass analysis.

Figure S2. Receiver operating characteristics (ROC) analysis for combined metabolites andmetabolites and lipoproteins subfraction variables between the different group comparision.

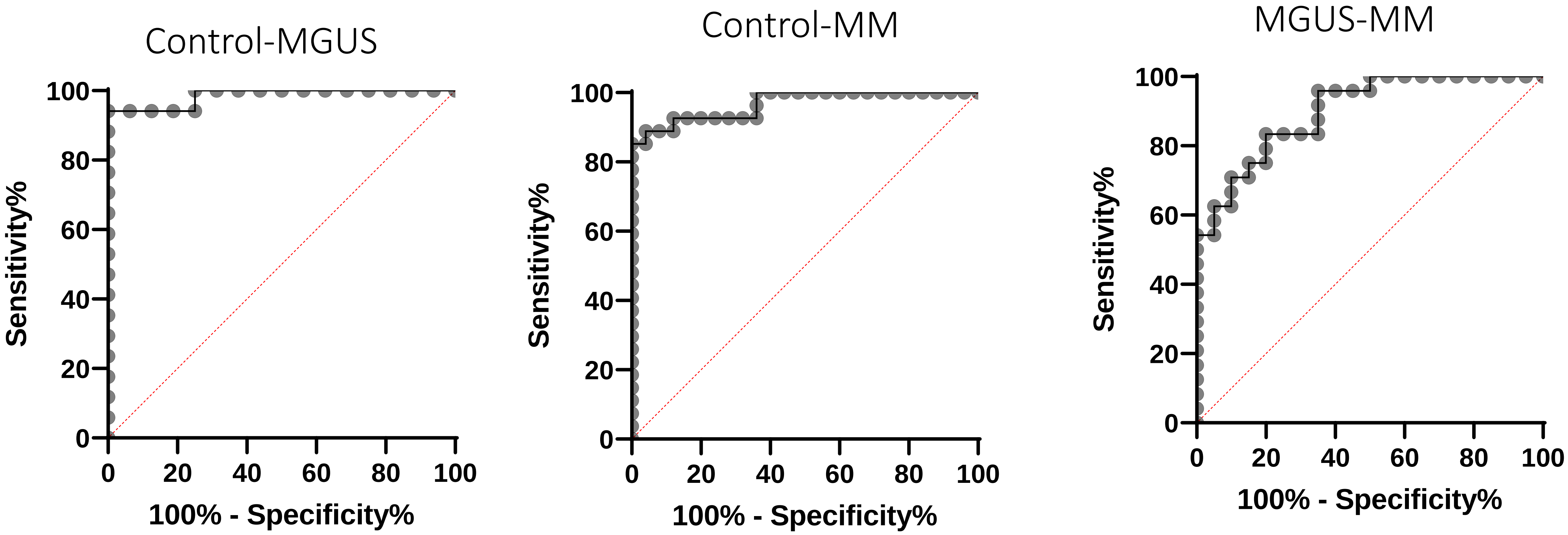

	Group	AUC	95% CI	SE	p-value
Lysine + Formic acid + Leucine + Alanine	Control-MGUS	0.985	0.952 – 1.000	0.017	1.98×10^{-6}
HDL 4-Cholesterol + HDL 4-Phospholipids + HDL 4-Apolipoprotein A1 + HDL 4-Apolipoprotein A2	Control-MM	0.967	0.925 – 1.000	0.021	7.54×10^{-9}
HDL Free cholesterol + Total Apolipoprotein A1 + HDL Apolipoprotein A1 + HDL 3-Cholesterol	MGUS-MM	0.900	0.813 – 0.986	0.044	6.02×10^{-6}

Figure S3. Correlation between clinical measurements, metabolites and lipoproteins subfraction variables

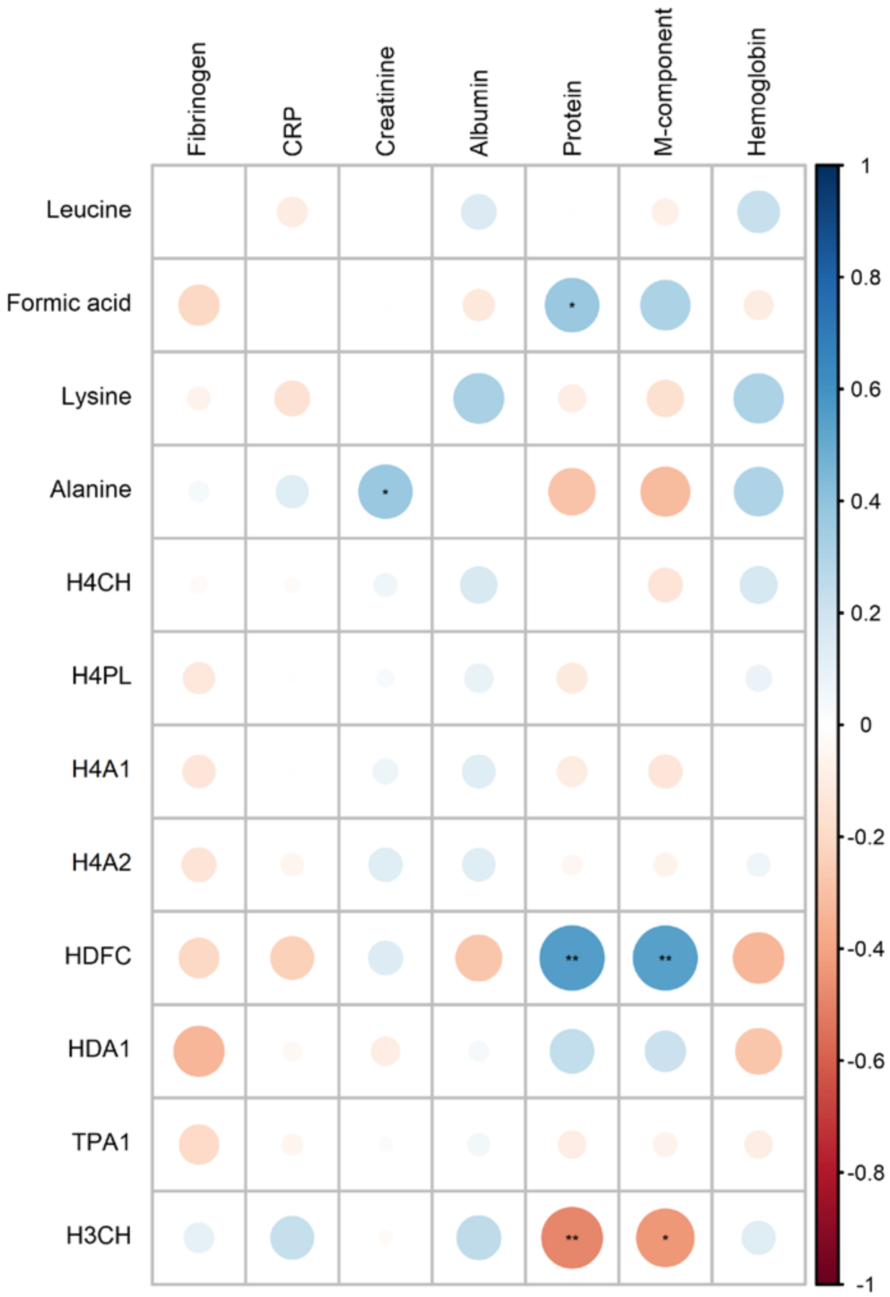

Figure S3: Correlation matrix displaying a positive/negative (blue/red) relationship between the clinical characteristics of MM patients and the lipoproteins. Significant correlations are shown using ***/**/*, which stand for p <0.001/<0.01/<0.05. H4CH: HDL-4 Cholesterol, H4PL: HDL-4 Phospholipids, H4A1: HDL-4 Apolipoprotein A-1, H4A2: HDL-4 Apolipoprotein A-2, HDFC: HDL Free Cholesterol, HDA1: HDL Apolipoprotein A-1, TPA1: Total Apolipoprotein A-1, and H3CH: HDL-3 Cholesterol.

Table s3. Healthy Control vs. MGUS. Diagnostic performance of metabolites and lipoprotein subfraction variables for Control vs. MGUS with VIP score ≥ 1.0. [a]*p*-values from Mann Whitney U Test. [b]FDR corrected *p*-values (*q*-value). [c] *p*-values from ROC analysis.

Metabolites [mmol/L or mg/dL]	Control	MGUS	FC	[a]p-value	[b]*q*-value	Sensitivity (%)	Specificity (%)	AUC (95% CI)	[c] *p*-value	VIP
	Median (min/max)	Median (min/max)								
Alanine	0.461 (0.327-0.715)	0.354 (0.220-0.572)	0.8	0.002	0.004	75	68	0.78 (0.64-0.91)	0.002	1.4
Glutamic acid	0.072 (0.045-0.140)	0.097 (0.064-0.156)	1.3	0.005	0.009	76.47	70	0.78 (0.63-0.93)	0.003	1.0
Isoleucine	0.059 (0.037-0.107)	0.045 (0.017-0.082)	0.8	<0.001	0.003	72.22	76	0.77 (0.63-0.91)	0.003	1.4
Leucine	0.099 (0.060-0.165)	0.071 (0.029-0.127)	0.7	<0.001	0.002	80	68	0.82 (0.69-0.94)	<0.001	1.6
Lysine	0.199 (0.146-0.287)	0.154 (0.154-0.079)	0.8	<0.001	0.001	94.12	70.83	0.86 (0.75-0.97)	<0.001	1.5
Methionine	0.080 (0.057-0.111)	0.073 (0.040-0.109)	0.9	0.040	0.041	57.89	76	0.66 (0.50-0.83)	0.066	1.0
Valine	0.233 (0.165-0.358)	0.200 (0.103-0.309)	0.9	0.020	0.025	75	52	0.7 (0.55-0.86)	0.020	1.1
Formic acid	0.025 (0.015-0.044)	0.040 (0.017-0.102)	1.6	<0.001	0.000	80	88.24	0.84 (0.71-0.97)	<0.001	1.7
Lactic acid	1.890 (1.394-4.980)	1.568 (0.742-2.806)	0.8	0.007	0.009	70	72	0.74 (0.59-0.88)	0.007	1.1
Acetone	0.020 (0.008-0.41)	0.038 (0.009-0.160)	1.9	0.005	0.009	60	78.26	0.72 (0.56-0.88)	0.013	1.2
Glycerol	0.232 (0.114-0.373)	0.234 (0.109-0.598)	1.0	0.003	0.007	62.5	50	0.52 (0.27-0.78)	0.854	1.2
HDL-4 Cholesterol	18.057 (13.593-24.054)	15.228 (5.628-23.353)	0.8	0.006	0.009	70	72	0.74 (0.59-0.89)	0.006	1.2
HDL-4 Phospholipids	25.969 (18.990-31.254)	22.647 (8.915-31.714)	0.9	0.032	0.036	70	60	0.69 (0.53-0.85)	0.032	1.1
HDL-4 Apolipoprotein-A2	17.029 (9.803-20.936)	14.748 (1.428-20.750)	0.9	0.040	0.041	70	56	0.68 (0.52-0.84)	0.040	1.0

Table S3. Diagnostic performance of metabolites and lipoprotein subfraction variables for Control vs. MGUS VIP score ≥ 1.0. [a] *p*-values from Mann Whitney U Test. [b]FDR corrected values. [c] *p*-values from ROC analysis.

Table S4. Healthy controls vs. MM. Diagnostic performance of metabolites and lipoprotein subfraction variables for Control vs. MM with VIP scores ≥ 1.0. [a] *p*-values from Mann Whitney U Test. [b]FDR corrected *p*-values (*q*-value). [c] *p*-values from ROC analysis.

Metabolites [mmol/L or mg/dL]	Controls	MM	FC	[a]*p*-value	[b]*q*-value	Sensitivity (%)	Specificity (%)	AUC (95% CI)	[c] *p*-value	VIP
	Median (min/max)	Median (min/max)								
Glutamine	0.638 (0.498-0.847)	0.462 (0.293-0.765	0.7	<0.001	<0.001	83.33	76.00	0.85 (0.74-0.95)	<0.001	1.0
Leucine	0.099 (0.060 – 0.165)	0.067 (0.035-0.115)	0.7	<0.001	<0.001	76.67	84.00	0.84 (0.74-0.95)	<0.001	1.0
Lysine	0.199 (0.146-0.287)	0.106 (0.039-0.260)	0.5	<0.001	<0.001	80.00	100.00	0.91 (0.81-1.00)	<0.001	1.1

Methionine	0.080 (0.057-0.111)	0.056 (0.033-0.090)	0.7	<0.001	<0.001	94.74	76.00	0.91 (0.82-0.99)	<0.001	1.2
Formic acid	0.025 (0.015-0.044)	0.040 (0.015-0.093)	1.6	<0.001	<0.001	73.33	70.59	0.79 (0.67-0.92)	<0.001	1.0
Total Cholesterol	178.307 (104.451-277.041)	112.233 (61.176-189.296)	0.6	<0.001	<0.001	83.30	84.00	0.91 (0.83-0.98)	<0.001	1.2
LDL Cholesterol	94.855 (47.060-176-295)	61.150 (9.824-107.420)	0.6	<0.001	<0.001	86.67	72.00	0.84 (0.73-0.94)	<0.001	1.0
HDL Cholesterol	56.129 (34.245-87.353)	35.017 (14.370-50.863)	0.6	<0.001	<0.001	83.30	72.00	0.89 (0.81-0.97)	<0.001	1.2
Total Apolipoprotein-A1	136.164 (91.824-171.295)	87.954 (45.471-145.260)	0.6	<0.001	<0.001	83.30	84.00	0.92 (0.86-0.99)	<0.001	1.3
Total Apolipoprotein-A2	28.819 (20.680-36.671)	19.195 (11.590-31.825)	0.7	<0.001	<0.001	93.30	68.00	0.89 (0.81-0.97)	<0.001	1.2
LDL Free Cholesterol	28.684 (18.178-51.147)	18.703 (4.560-32.269)	0.7	<0.001	<0.001	80.00	72.00	0.85 (0.75-0.94)	<0.001	1.0
HDL Free Cholesterol	13.660 (8.142-23.157)	5.480 (0.016-12.821)	0.4	<0.001	<0.001	80.77	100.00	0.94 (0.89-1.00)	<0.001	1.3
HDL Phospholipids	76.233 (49.182-111.248)	53.707 (30.603-80.644)	0.7	<0.001	<0.001	86.67	72.00	0.86 (0.76-0.96)	<0.001	1.1
HDL Apolipoprotein-A1	138.692 (92.951-176.117)	89.671 (35.903-143.393)	0.6	<0.001	<0.001	86.67	84.00	0.92 (0.84-0.99)	<0.001	1.2
HDL Apolipoprotein-A2	28.611 (21.542-37.212)	19.836 (13.875-33.474)	0.7	<0.001	<0.001	76.67	84.00	0.88 (0.79-0.97)	<0.001	1.1
VDL-5 Cholesterol	1.648 (0.272-2.453)	0.610 (0.152-2.954)	0.4	<0.001	<0.001	83.30	88.00	0.82 (0.67-0.97)	<0.001	1.1
VDL-3 Phospholipids	2.494 (0.280-5.506)	4.782 (2.063-8.791)	1.9	<0.001	<0.001	80.00	72.00	0.86 (0.77-0.96)	<0.001	1.0
LDL-4 Free Cholesterol	3.889 (1.311-7.709)	1.987 (0.151-5.199)	0.5	<0.001	<0.001	87.50	68.00	0.83 (0.72-0.94)	<0.001	1.1
HDL-3 Cholesterol	10.080 (6.716-13.449)	5.360 (1.380-9.295)	0.5	<0.001	<0.001	73.30	100.00	0.94 (0.88-0.99)	<0.001	1.3
HDL-4 Cholesterol	18.057 (13.593-24.054)	8.841 (1.818-14.359)	0.5	<0.001	<0.001	92.59	100.00	0.99 (0.98-1.0)	<0.001	1.4

HDL-4 Free Cholesterol	3.479 (1.927-4.627)	2.267 (0.699-3.649)	0.7	<0.001	<0.001	80.77	68.00	0.83 (0.72-0.94)	<0.001	1.0
HDL-4 Phospholipids	25.969 (18.990-31.254)	14.391 (3.770-22.178)	0.6	<0.001	<0.001	96.55	96.00	0.99 (0.98-1.0)	<0.001	1.4
HDL-2 Apolipoprotein-A1	19.105 (11.500-26.569)	12.445 (5.824-18.980)	0.7	<0.001	<0.001	90.00	72.00	0.86 (0.76-0.96)	<0.001	1.1
HDL-3 Apolipoprotein-A1	27.351 (16.606-35.961)	16.100 (7.834-27.803)	0.6	<0.001	<0.001	83.30	80.00	0.90 (0.82-0.98)	<0.001	1.2
HDL-4 Apolipoprotein-A1	67.912 (48.438-80.946)	36.984 (6.700-54.699)	0.5	<0.001	<0.001	93.10	92.00	0.99 (0.96-1.0)	<0.001	1.4
HDL-4 Apolipoprotein-A2	17.029 (9.803-20.936)	10.241 (2.4-14.434)	0.6	<0.001	<0.001	96.55	92.00	0.96 (0.92-1.0)	<0.001	1.3

Table S5. MGUS vs. MM. Diagnostic performance of metabolites and lipoprotein subfraction variables for MGUS vs. MM with VIP scores ≥ 1.0. [a] *p*-values from Mann Whitney U Test. [b]FDR corrected *p*-values (*q*-value). [c] *p*-values from ROC analysis.

Metabolites [mmol/L or mg/dL]	MGUS	MM	FC	[a]p-value	[b]q- value	Sensitivity (%)	Specificity (%)	AUC (95% CI)	[c] p-value	VIP
	Median (min/max)	Median (min/max)								
Glutamine	0.632 (0.347-0.937)	0.462 (0.293-0.765)	0.7	<0.001	<0.001	80	70	0.79 (0.66-0.92)	<0.001	1.1
Total Cholesterol	175.108 (105.713-307.523)	112.233 (61.176-189.296)	0.6	<0.001	<0.001	80	75	0.86 (0.75-0.96)	<0.001	1.3
HDL-Cholesterol	51.403 (31.261-91.616)	35.017 (14.370-60.863)	0.7	<0.001	<0.001	86.67	75	0.86 (0.75-0.97)	<0.001	1.3
Total Apolipoprotein-A1	127.682 (90.308-215.506)	87.954 (45.471-145.260)	0.7	<0.001	<0.001	90	85	0.92 (0.84-0.99)	<0.001	1.4
Total Apolipoprotein-A2	26.175 (16.906-40.164)	19.195 (11.590-31.825)	0.7	<0.001	<0.001	70	80	0.80 (0.68-0.92)	<0.001	1.1
LDL Free Cholesterol	28.700 (10.758-55.213)	18.703 (4.560-32.269)	0.7	<0.001	<0.001	76.67	70	0.79 (0.67-0.92)	<0.001	1.1
HDL Free Cholesterol	13.231 (3.825-31.399)	5.480 (0.016-12.821)	0.4	<0.001	<0.001	96.15	80	0.93 (0.86-1.0)	<0.001	1.5
HDL Phospholipids	72.849 (44.936-129.936)	53.707 (30.603-80.644)	0.7	<0.001	<0.001	83.3	80	0.84 (0.71-0.96)	<0.001	1.2

HDL Apoliprotein-A1	127.673 (87.859-219.151)	89.671 (35.903-143.393)	0.7	<0.001	<0.001	90	80	0.90 (0.81-0.99)	<0.001	1.3
LDL-3 Free Cholesterol	3.978 (0.500-10.358	2.550 (0.171-5.132)	0.6	<0.001	<0.001	77.78	75	0.77 (0.63-0.91)	0.0018	1.0
LDL-4 Free Cholesterol	3.659 (1.860-7.811)	1.987 (0.151-5.199)	0.5	<0.001	<0.001	70.83	78.95	0.84 (0.72.-0.96)	<0.001	1.2
HDL-3 Cholesterol	8.824 (5.866-15.083)	5.360 (1.380-9.295)	0.6	<0.001	<0.001	70	85	0.89 (0.80-0.97)	<0.001	1.3
HDL-4 Cholesterol	15.228 (5.628-23.353)	8.841 (1.818-14.359)	0.6	<0.001	<0.001	85.19	80	0.87 (0.77-0.98)	<0.001	1.0
HDL-1 Free Cholesterol	4.973 (0.536-17.467)	2.094 (0.320-5.183)	0.4	<0.001	<0.001	74.07	80	0.81 (0.69-0.94)	<0.001	1.1
HDL-2 Free Cholesterol	1.947 (0.850-3.879)	1.379 (0.140-2.816)	0.7	0.001	<0.001	89.66	70	0.76 (0.61-0.92)	0.017	1.0
HDL-4 Free Cholesterol	3.144 (0.881-5.028)	2.267 (0.699-3.649)	0.7	0.001	0.0010448	80.77	60	0.74 (0.60-0.89)	0.0052	1.0
HDL-4 Phospholipids	22.647 (8.915-31.714)	14.391 (3.770-22.178)	0.6	<0.001	<0.001	96.55	75	0.87 (0.76-0.99)	<0.001	1.3
HDL-2 Apolipoprotein-A1	17.965 (9.071-32.481)	12.445 (5.824-18.980)	0.7	<0.001	<0.001	73.3	80	0.81 (0.68-0.94)	<0.001	1.2
HDL-3 Apolipoprotein-A1	24.218 (15.842-37.091)	16.100 (7.834-27.803)	0.7	<0.001	<0.001	73.3	80	0.84 (0.74-0.95)	<0.001	1.2
HDL-4 Apolipoprotein-A1	60.351 (25.041-78.275)	36.984 (6.700-54.699)	0.6	<0.001	<0.001	89.66	85	0.88 (0.77-0.99)	<0.001	1.4
HDL4 Apolipoprotein-A2	14.748 (1.428-20.750)	10.241 (2.400-14.434)	0.7	<0.001	<0.001	93.3	70	0.79 (0.64-0.93)	<0.001	1.0

Figure S4. Priniciple component analysis of Control versus MGUS, Control versus MM and MGUS versus MM

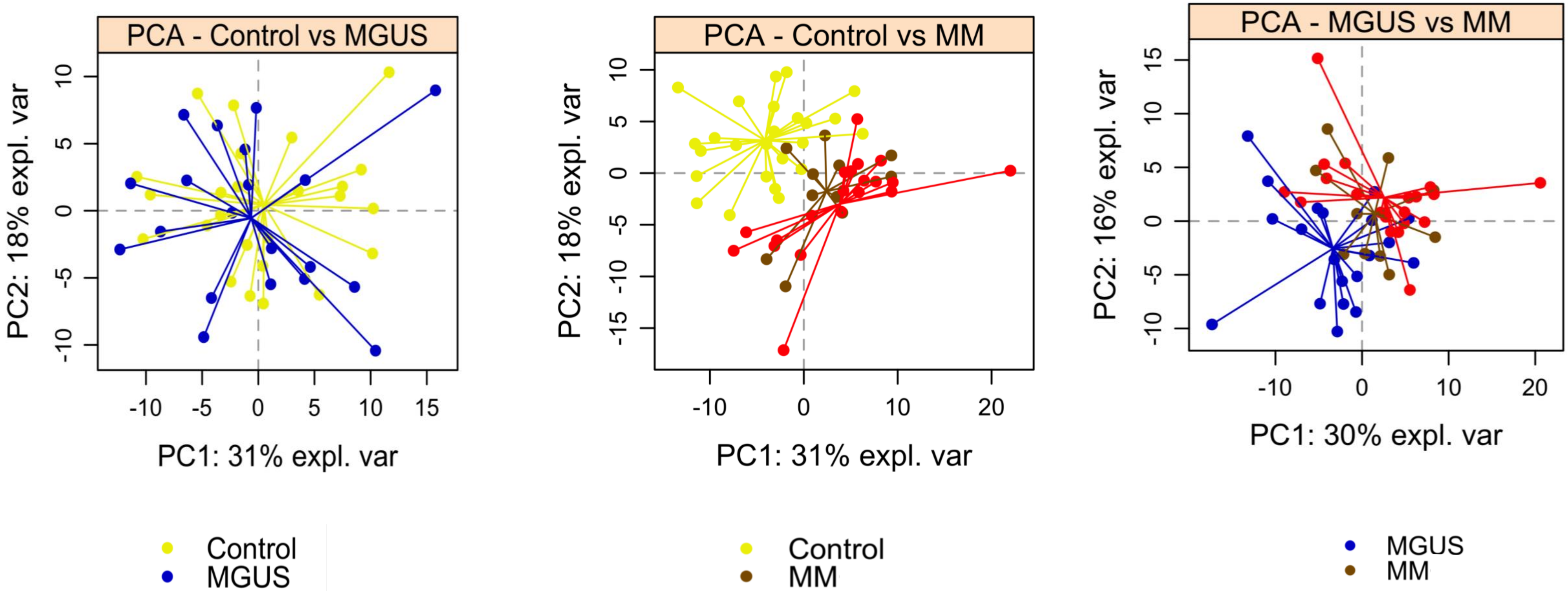

Supplement: Supplementary file 1 [file ijms-24-12275-s001.zip › Supplementary File S2.pdf]
